# Pediatric Sjögren’s Disease: Literature Review and Diagnostic Challenges in an Uncommon Case

**DOI:** 10.3390/diagnostics16121926

**Published:** 2026-06-22

**Authors:** Otilia Elena Frăsinariu, Dragoș Florin Teșoi, Anca Cardoneanu, Ileana Katerina Ioniuc, Ana Maria Scurtu, Elena Cojocaru, Larisa Ioana Teșoi, Ionut Daniel Iancu, Anamaria Laura Buga, Ingrith Crenguța Miron

**Affiliations:** Grigore T. Popa University of Medicine and Pharmacy, 700115 Iasi, Romania; frasinariu.otilia@umfiasi.ro (O.E.F.); ingridmiron@gmail.com (I.C.M.)

**Keywords:** childhood-onset Sjögren’s disease, recurrent parotitis, salivary gland ultrasonography, sicca symptoms, extraglandular manifestations

## Abstract

**Background and Clinical Significance:** Childhood-onset Sjögren’s disease (cSjD) is a rare autoimmune disorder that remains challenging to diagnose because of its heterogeneous clinical presentation and the frequent absence of classic sicca symptoms at disease onset. Recurrent parotitis and systemic manifestations often predominate in pediatric patients, contributing to diagnostic delay and potential irreversible glandular damage. Early recognition is essential to prevent complications and improve long-term outcomes. **Case Presentation:** We report the case of a 17-year-old female diagnosed with primary Sjögren’s disease following a prolonged history of recurrent parotid involvement and progressive glandular dysfunction. Comprehensive evaluation revealed positive anti-SSA antibodies, hypergammaglobulinemia, characteristic salivary gland ultrasonography abnormalities, and a positive minor salivary gland biopsy, resulting in fulfillment of all domains of the 2016 ACR/EULAR classification criteria. The patient also exhibited unusual vascular findings, including carotid atheromatous calcifications in the absence of traditional cardiovascular risk factors. **Conclusion:** This case highlights the diagnostic complexity of cSjD and underscores the value of a multimodal diagnostic approach integrating clinical assessment, serology, imaging, and histopathology. The presence of early vascular abnormalities broadens the spectrum of potential extraglandular manifestations and emphasizes the need for comprehensive evaluation and long-term monitoring in affected patients.

## 1. Introduction

Sjögren’s disease (SjD) is a systemic autoimmune condition marked by lymphocytic infiltration of exocrine glands. It particularly affects the lacrimal and salivary glands, which results in sicca symptoms. However, in pediatric populations, the disease often shows atypical signs and differs significantly from adult cases [1,2,3].

Children with SjD typically do not present with the classic glandular manifestations seen in adults. They more frequently develop recurrent parotitis, systemic features, or non-specific complaints [3,4]. Consequently, due to the infrequent reporting of dryness symptoms in young children, the disease is often misdiagnosed as infectious parotitis, which delays the correct diagnosis [2,3,4,5].

Recent large studies of pediatric cases show that symptoms typically start at around 9 to 12 years of age, although earlier signs may appear. The condition is more common in girls, but boys can also be affected [3,4]. Common serological markers include anti-Ro/SSA and anti-La/SSB antibodies, antinuclear antibodies (ANAs), rheumatoid factor, and elevated immunoglobulin levels, although these are not universally present. Imaging techniques, such as salivary gland ultrasound, along with objective assessments of gland function or biopsy, are increasingly utilized [3,4].

The delay in diagnosing cSjD has serious consequences. It can lead to ongoing inflammation, greater glandular damage, a higher risk of extraglandular manifestations, unnecessary antibiotic use due to suspected infectious parotitis, and delays in starting immunomodulatory treatment. Therefore, it is crucial to increase awareness of cSjD in cases of chronic parotitis that do not respond to standard treatment.

We report a case of pediatric primary SjD in a 17-year-old female, characterized by recurrent parotitis, cervical lymphadenopathy, and inability to cry, highlighting the diagnostic complexity and the importance of considering cSjD in the differential diagnosis of chronic parotid disease in children and adolescents. The novelty of this case lies in the unusually early systemic involvement, prolonged diagnostic delay despite objective inflammatory markers, and evidence suggesting early vascular dysfunction in cSjD, features that remain insufficiently characterized in pediatric cohorts. Importantly, the presence of early vascular-related findings in this patient supports the emerging hypothesis that immune-mediated endothelial dysfunction may represent an underrecognized component of cSjD pathogenesis. Given the diagnostic complexity of cSjD and the heterogeneity of reported presentations, we complemented this case description with a focused narrative literature review to contextualize our findings and highlight recurring diagnostic pitfalls. Relevant articles published between 2000 and 2025 were identified through searches in PubMed, Scopus, and Web of Science databases using combinations of the following keywords: “juvenile Sjögren’s disease”, “pediatric Sjögren’s disease”, “primary Sjögren’s syndrome in children”, “autoimmunity”, “extraglandular manifestations”, and “vascular involvement”. Articles were selected based on their relevance to clinical presentation, diagnostic challenges, pathophysiological mechanisms, and systemic manifestations in pediatric patients. Additional references were identified through manual screening of the bibliographies of selected articles. As this review was intended to provide clinical context for the presented case rather than to constitute a systematic review, no formal study selection framework or quality assessment was applied.

### 1.1. Epidemiology

cSjD presenting in childhood is rare, representing only a small fraction of all SjD cases. Quantifying its true frequency is challenging due to evolving diagnostic criteria, the absence of pediatric-specific criteria, and the overlap in published case reports and cohorts. The earliest and most comprehensive literature review, published in 2003, identified 253 pediatric cases documented since the initial descriptions of SjD [6]. Subsequent decades have yielded additional case reports and small case series, along with the first multicenter pediatric cohorts. However, since many of these post-2003 cases are integrated into overlapping reviews or included in registry studies, no later publication has produced a verified, de-duplicated cumulative count of all cSjD cases.

Large-scale international registries have offered valuable contemporary estimates. In the multinational Big Data Sjögren Project, only 158 out of 12,083 patients (1.3%) had disease onset before age 19. This represents the largest uniformly characterized cohort of pediatric-onset cases to date, confirming that cSjD constitutes only a small portion of all primary SjD cases. In this registry-based cohort, the childhood group exhibited a significant female predominance (136 out of 158), a mean age at diagnosis of approximately 14.2 years, high rates of autoantibody positivity (about 90% for ANAs and 89% for anti-Ro/La), frequent abnormal minor salivary gland biopsies, and common systemic activity, particularly in glandular involvement and lymphadenopathy, as measured by the ESSDAI domains. These registry data represent one of the most robust and consistently characterized samples of pediatric cases to date [7].

An international retrospective workgroup gathered cases from 23 centers across eight countries and identified 300 children diagnosed with SjD (aged under 18 years). This large cohort highlighted two significant issues:

- recurrent or persistent parotitis and arthralgia were common non-sicca presentations;
- most children in the dataset did not fulfill the adult 2016 ACR/EULAR classification criteria when applied retrospectively, with only a minority meeting the criteria and many lacking complete testing.

These findings underscore both the systemic nature of pediatric disease and the limitations of adult-derived criteria when applied to children [3].

Together, these data emphasize that cSjD, although rare, often presents with a systemic phenotype distinct from the classic adult presentation, and that current criteria may lack sensitivity for this population.

### 1.2. Pathophysiology

cSjD is driven by immune-mediated mechanisms similar to those described in adults, although disease onset may occur earlier and systemic manifestations may be more prominent in children. Genetic susceptibility involving HLA and immune-regulatory loci interacts with environmental or viral triggers. These factors activate glandular epithelial cells and initiate a type-I interferon (IFN-I) response in many patients [8,9].

Salivary and lacrimal gland epithelial cells actively contribute to local inflammation. They produce B-cell activating factor (BAFF), chemokines such as CXCL13, and IFN-inducible genes. These mediators recruit plasmacytoid dendritic cells and amplify immune activation within the glands [8,9,10].

B-cell hyperactivity represents a central pathogenic mechanism in SjD. Elevated BAFF levels and expansion of naïve and memory B-cell subsets promote autoantibody production, including anti-SSA/Ro and anti-SSB/La, and contribute to ectopic germinal-center–like structures within salivary glands. Alterations in T follicular helper and peripheral helper T-cell subsets further sustain B-cell–driven immune activation. Similar patterns of immune dysregulation have been described in pediatric cohorts [8,11].

Local lymphocytic infiltration, also known as focal lymphocytic sialadenitis, leads to progressive injury of acinar and ductal epithelial cells, resulting in apoptosis and a gradual decline in secretory function. Over time, chronic inflammation promotes fibrosis and fatty replacement of glandular tissue, which contributes to the delayed onset of sicca symptoms frequently observed in pediatric patients. Systemic manifestations—including hematologic, renal, cutaneous, and neurological involvement—are thought to result from immune-complex deposition and inflammatory processes extending beyond the exocrine glands [8,12].

From a practical standpoint, the activation of the IFN-I axis and the BAFF-driven B-cell actions represent quantifiable pathway signatures and potential therapeutic targets. Recent research advocates for the integration of IFN and B-cell biomarkers into patient stratification and clinical trial design. Nevertheless, data specific to pediatric populations remain limited, and further studies are needed to clarify age-related differences in immune mechanisms and clinical expression [10,11,12].

### 1.3. Clinical Features

The clinical spectrum of cSjD differs from that of adult disease. In children, recurrent glandular swelling and systemic manifestations commonly predominate, whereas classic sicca symptoms may be less prominent at the first presentation, compared with adults. Large multicenter registry studies and pediatric cohorts show a variety of presentations. For instance, in the Sjögren Big Data analysis of 158 childhood-onset cases, approximately 80% reported xerostomia and around 70% experienced dry eyes. Parotid enlargement occurred in about 33%, and 96.7% of pediatric patients had positive findings in minor-salivary-gland biopsies, indicating a significant presence of glandular involvement [7]. Looking at single-center pediatric studies, some variations can arise based on age and referral patterns. For example, a Chinese pediatric cohort of 39 patients noted glandular enlargement or recurrent parotitis in about 25.6%, dry mouth or eyes in 23.1%, and systemic involvement in 56.4% of patients [2]. Another Chinese study involving 49 patients reported a mean age at diagnosis of approximately 10.3 years, with a female-to-male ratio of about 6/1. In this study, skin involvement was the most commonly observed manifestation in children with SjD. This sign was reported in 51% of patients, most often manifesting as purpura, and was followed by parotitis, which was present in 36.7% of cases [13].

Common extraglandular features reported in pediatric cases include cutaneous manifestations (such as rash or purpura), fever, and lymphadenopathy. Although rare, significant complications—including interstitial lung disease and neurological symptoms that can mimic demyelinating disorders—have also been documented [1,2,14].

Renal involvement in cSjD is relatively uncommon but often more clinically significant than in adults. The most frequent manifestation is tubulointerstitial nephritis leading to distal renal tubular acidosis (dRTA), which may present with growth impairment, recurrent vomiting, polyuria, muscle weakness, nephrocalcinosis, or persistent electrolyte abnormalities—especially hypokalemia and metabolic acidosis. Some children may initially present with dRTA before any glandular manifestations, making renal disease an important early diagnostic clue. Glomerular disease is uncommon in children with SjD, but remains an important part of the differential when renal findings extend beyond tubular dysfunction. Reported manifestations include proteinuria, microscopic hematuria, and occasional immune-complex glomerulonephritis associated with systemic autoimmunity or low complement levels. A notable reported case describes an 8-year-old girl who initially presented with nephrotic-range proteinuria and hypoalbuminemia due to membranous nephropathy. This aspect led to the diagnosis of SjD after sicca features and lymphocytic salivary gland infiltration were identified. Across cohorts, renal involvement in cSjD tends to reflect a more active autoimmune phenotype, often requiring early recognition and targeted immunosuppression to prevent chronic kidney disease [1,15,16,17].

cSjD often scores higher across various systemic ESSDAI domains (such as glandular, constitutional, lymphadenopathy, cutaneous, and hematological) compared with adults, emphasizing the tendency for multisystem involvement in younger patients [7].

Because estimates can vary widely based on factors such as referral sources, geographic locations, and diagnostic practices, clinicians need to interpret these findings carefully and remain vigilant. All these studies emphasize how important it is to maintain a high level of suspicion of SjD in children who present with repeated episodes of parotid swelling or multisystem inflammatory symptoms [1,2,7,13,14].

### 1.4. Diagnosis

The diagnosis of cSjD is challenging due to the lack of validated pediatric-specific criteria and the atypical presentation in children. A recent single-center study involving 54 children found that classic sicca symptoms, such as dry mouth and dry eyes, were reported at low frequencies (13% and 9.3%, respectively), even though a high percentage of the children tested positive for anti-Ro/SSA antibodies (94.4%). Salivary gland functional testing showed abnormal results in 70.4% of cases, and ultrasound results were abnormal in 55.6%, indicating glandular involvement [4]. The commonly used adult classification criteria—the 2016 ACR/EULAR Classification Criteria for primary Sjögren’s syndrome (pSS)—do not fully apply to children, as they emphasize symptoms of glandular dysfunction more typically seen in adults [4,18].

Diagnosing and evaluating pediatric SjD requires a multimodal, stepwise approach, as subjective sicca complaints are frequently absent. The pediatric phenotype may instead feature glandular swelling and other systemic manifestations. First-line laboratory tests should include ANA profile, anti-SSA/Ro and anti-SSB/La antibodies, rheumatoid factor (RF), quantitative immunoglobulins, complement levels, and routine baseline complete blood count (CBC), renal, and liver function tests to screen for common extraglandular involvement and therapy-related abnormalities [3,4]. In children experiencing recurrent infections or growth failure, consider adding immunodeficiency screening to rule out primary immune defects that may mimic or coexist with SjD [13].

Since children often underreport dryness, objective ocular testing (such as Schirmer test, ocular surface staining, van Bijsterveld, or the Oxford score) and measurement of salivary flow (unstimulated whole saliva when feasible) should be conducted early in the evaluation. Abnormal objective ocular or salivary tests provide stronger diagnostic support in pediatric cohorts than subjective symptoms alone [4]. While salivary flow rates may not correlate perfectly with symptoms, they provide functional data that complement imaging and serology findings. Recent studies emphasize the importance of pairing Schirmer tests and ocular staining with salivary function tests in children suspected of having cSjD [4,19].

Salivary gland ultrasonography (SGUS) has become a key investigation that is non-invasive and child-friendly. SGUS can detect characteristic heterogeneous, hypoechoic changes, glandular inhomogeneity, and loss of normal gland architecture. It also allows for semi-quantitative scoring (OMERACT/SGUS score) that correlates with histopathology and serological activity in both pediatric and adult cohorts [20,21]. In several pediatric series, SGUS abnormalities were found in over 50% of affected children, assisting in identifying candidates for biopsy or further functional imaging [4,21]. Ultra-high-frequency ultrasonography (UHF-US) and improvements in SGUS scoring are promising for enhancing sensitivity in evaluating smaller glands in children [22].

When SGUS and functional tests yield inconclusive results, salivary-gland functional imaging (scintigraphy) can document uptake and excretion deficits and may provide complementary functional information to structural ultrasound, particularly in seronegative or discordant cases [19,23]. Cross-sectional imaging (such as MRI/CT) is indicated for atypical, asymmetric, or mass-forming lesions to rule out obstructive sialadenitis, abscesses, or neoplasms before any invasive procedures are performed. Additional imaging techniques that may complement salivary gland ultrasonography and conventional magnetic resonance imaging include MR sialography, multidetector computed tomography (MDCT) sialography, and cone-beam computed tomography (CBCT) sialography. MR sialography is a non-invasive technique that does not require contrast administration and allows detailed visualization of the salivary ductal system using heavily T2-weighted sequences. It has demonstrated good sensitivity for detecting ductal dilatation, sialectasia, and structural glandular abnormalities associated with SjD. Because it avoids ionizing radiation, MR sialography may be particularly attractive in pediatric patients requiring repeated evaluations. However, its routine use may be limited by cost, availability, and longer acquisition times. MDCT sialography provides high-resolution anatomical assessment of both the salivary ducts and glandular parenchyma and can identify stenosis, ductal irregularities, and sialectatic changes. Nevertheless, the associated radiation exposure restricts its application in children and adolescents. More recently, CBCT sialography has emerged as a promising three-dimensional imaging modality capable of providing excellent spatial resolution and detailed visualization of peripheral salivary ducts. Compared with conventional two-dimensional sialography, CBCT sialography offers improved characterization of ductal abnormalities and intraglandular structural changes while facilitating three-dimensional assessment of the salivary gland architecture. Characteristic findings described in SjD include multiple areas of punctate or globular sialectasia producing a “cherry blossom” appearance. Comparative studies suggest that both MR sialography and CBCT sialography may serve as valuable adjunctive diagnostic tools in the evaluation of salivary gland disorders, with MR sialography showing particular advantages for the detection of ductal dilatations and non-calcified lesions. Although evidence in pediatric SjD remains limited, these advanced imaging modalities may provide additional diagnostic information in selected cases when ultrasonography findings are inconclusive or when a more detailed assessment of salivary gland architecture is required [24,25,26,27].

Minor salivary-gland biopsy (mSGB) remains the most specific single diagnostic test when positive. It is especially useful in seronegative children or when a definitive diagnosis is necessary to guide management. Reported sensitivity and specificity vary by study, but recent evaluations indicate sensitivity in the range of approximately 64–94% and specificity of 61–100%, depending on the focus-score thresholds and sampling techniques used [20,28]. Due to its invasive nature, the indications for biopsy in children should be carefully targeted; this includes cases that are seronegative with objective gland dysfunction, atypical presentations, suspicion of alternative histological entities (such as IgG4-related disease or sarcoidosis), or when lymphoma is suspected. Emerging less-invasive methods that combine SGUS with serological assessments may achieve diagnostic accuracy approaching that of biopsy in selected populations, but biopsy remains the reference standard in uncertain cases [19].

Complementary laboratory and imaging assessments for extraglandular disease should be guided by clinical features:

- urinalysis and urine protein testing, along with renal function tests for suspected renal involvement;
- chest imaging and pulmonary function tests for respiratory symptoms;
- neuroimaging and cerebrospinal fluid testing for neurological signs;
- skin or nerve biopsies for vasculitic features when indicated [3,4].

In practice, a comprehensive pediatric investigation bundle should include: patient history and examination, tests for ANAs, SS-A and SS-B antibodies, RF, CBC, and renal and hepatic profiles, as well as Schirmer testing, ocular staining, unstimulated salivary flow measurement, SGUS, targeted infection and immunodeficiency tests, and selective mSGB when specific diagnostic information is needed [3,4,13,19,20].

### 1.5. Management

Management of cSjD is largely extrapolated from adult experience and centers on a multidisciplinary approach involving pediatric rheumatology, ophthalmology, dentistry, and other specialties as indicated [29]. Patient and parent education is essential, with emphasis on oral hygiene, saliva substitutes, ocular lubricants, and preventive dental care [29]. Topical therapy remains the first-line treatment for sicca symptoms, including artificial tears, tear-conserving measures, and topical anti-inflammatory eye drops for keratoconjunctivitis sicca [29].

Management of cSjD remains largely empirical because high-quality pediatric trials are scarce. A recent systematic review highlighted that therapy is frequently extrapolated from adult SjD treatment [29]. According to a survey of pediatric rheumatologists, the most used systemic therapies in practice are hydroxychloroquine, corticosteroids, methotrexate, rituximab, and mycophenolate mofetil, particularly for organ-threatening or extraglandular manifestations [30]. For systemic complications, biologic therapy such as rituximab is used off-label in cSjD, especially in the context of renal or neurological involvement, though consensus remains limited and practice varies [29,31]. In addition, there is growing support for sialendoscopy in children with recurrent glandular swelling, with longitudinal data showing symptomatic benefits and improved gland appearance on salivary gland ultrasound [32].

Given the lack of standardized guidelines, the British Society for Rheumatology consensus (which includes juvenile-onset cases) recommends short-term systemic steroids for specific organ complications and reserves conventional immunosuppressants for severe disease, as risks may outweigh benefits [33]. Close monitoring with disease activity scales, serologies, and non-invasive tools such as SGUS is encouraged, and tailored, multidisciplinary care (rheumatology, nephrology, ophthalmology) is essential.

### 1.6. Differential Diagnosis

The differential diagnosis for SjD is extensive and should be systematically approached by considering various categories: gland-directed causes, systemic autoimmune disorders, infectious diseases, congenital or metabolic issues, post-transplant or immune deficiency complications, and tumors. Key distinguishing features include the pattern of gland involvement (e.g., recurrent, unilateral or bilateral, painful or painless), the presence of systemic signs (such as fever, rash, or lymphadenopathy), and assessments through serology, imaging, and histopathology. A structured algorithmic approach that integrates clinical history, targeted serology, salivary gland ultrasound (and sialography or sialendoscopy when necessary), focused infectious testing, and potentially a minor salivary gland biopsy can help optimize the differentiation between these conditions [34].

Juvenile recurrent parotitis (JRP) is a primary alternative diagnosis for children with recurrent salivary gland swelling. In these cases, the typical onset is at a younger age, episodes are non-suppurative, and patients commonly show improvement with conservative measures or sialendoscopy. These characteristics differentiate JRP from SjD. Obstructive sialadenitis or sialolithiasis typically causes painful, meal-related swelling and can be identified through ultrasound or sialendoscopy [35,36]. Other autoimmune diseases—such as systemic lupus erythematosus, juvenile idiopathic arthritis with secondary sicca presentation, mixed connective tissue disease, and systemic sclerosis—can also lead to glandular manifestations or multisystem involvement. These conditions can be distinguished by disease-specific autoantibodies, unique systemic organ signatures, and distinct histological features. IgG4-related disease (IgG4-RD), which can affect the salivary and lacrimal glands, typically presents as painless, often symmetrical enlargement with storiform fibrosis, IgG4-positive plasma cell infiltrates on biopsy, and elevated serum IgG4 levels in many cases. Histological analysis and IgG4 staining are necessary to differentiate IgG4-RD from SjD. Sarcoidosis should also be considered, particularly if non-caseating granulomas are observed or if systemic granulomatous features are present [37,38,39]. Additionally, cystic fibrosis and other causes of exocrine dysfunction may manifest with oral dryness or recurrent infections accompanied by salivary abnormalities. In such scenarios, iontophoresis and CFTR genetic testing can serve as effective diagnostic aids when the clinical context suggests cystic fibrosis [40,41]. Endocrine issues, such as uncontrolled diabetes mellitus, as well as dehydration or medication-induced sicca caused by anticholinergics, psychotropic drugs, or antihistamines, should also be reviewed in the patient’s medication and systemic history. Persistent, asymmetric, rapidly progressive, or hardened gland masses might raise suspicion of salivary gland neoplasia, including MALT lymphoma, particularly in adolescents or in glands atypical for JRP. Such cases necessitate prompt imaging and excisional biopsy. While lymphoma is rare in cSjD, it is essential to remain vigilant in the presence of alarm features such as B symptoms, rapid enlargement, or a refractory course [42].

Differentiating cSjD from infectious parotitis relies on recognized differences in clinical patterns, laboratory findings, and imaging results. Infectious parotitis, such as mumps or bacterial sialadenitis, usually presents with an acute onset of painful, erythematous, and tender parotid swelling often accompanied by fever, with improvement after targeted antimicrobial or antiviral therapy. In contrast, cSjD typically manifests as recurrent or persistent parotid swelling that is usually painless and evolves over weeks or months rather than days [1,43,44,45]. Laboratory testing supports a diagnosis of SjD when there are positive ANAs and anti-SSA/SSB antibodies or hypergammaglobulinemia. In comparison, infectious causes are indicated by elevated acute inflammatory markers and pathogen-specific serology or PCR [1,45]. Moreover, salivary gland ultrasound can further assist in differentiation, with SjD showing heterogeneous parenchyma featuring small hypoechoic foci, contrasting with diffuse gland enlargement or focal abscess formation typically seen in acute infections [46,47]. Failure to improve with antimicrobial treatment, the presence of systemic autoimmune features (such as arthralgia or cytopenias), or recurrent episodes should prompt rheumatologic evaluation, which may include salivary gland ultrasound and/or a salivary gland biopsy for suspected cSjD [1,43].

The differential diagnosis for suspected cSjD is extensive. A methodical, evidence-based approach that combines clinical pattern recognition with targeted serology, imaging, and selective biopsy will effectively differentiate SjD from other conditions. In cases where uncertainty persists, longitudinal follow-up and repeat testing are often necessary, as pediatric presentations may evolve, and serological or histological markers may appear later in the disease course [34].

### 1.7. Prognosis

The long-term prognosis of cSjD is not completely understood and appears to be variable. Many children experience a chronic, relapsing course characterized by ongoing glandular disease and intermittent systemic flares. In some cases, a subset of children may develop progressive involvement of extraglandular organs, leading to increased morbidity [2,4,13]. Published studies of pediatric cohorts indicate that children often continue to experience glandular swelling along with a significant burden of systemic manifestations, including hematological, renal, hepatic, and cutaneous issues during follow-up. Some studies have documented persistent objective gland dysfunction based on imaging and tests, even when children report limited subjective dryness symptoms at presentation [2,4,13]. In adults, predictors of a more complicated disease course include factors such as persistent parotid swelling, cryoglobulinemia or hypergammaglobulinemia, low complement levels, and lymphadenopathy. These predictors have been associated with a heightened risk of lymphoma and worse outcomes. While lymphoma is rare in children, these adult risk indicators inform pediatric vigilance and have been applied cautiously when present [48,49]. Recent pediatric research highlights the need for structured outcome measures and long-term registries, such as the Florida Scoring System and ESSDAI adaptations. Limitations in sample sizes and variability in follow-up have hindered robust prognostication. Early efforts to stratify children at higher risk of systemic disease and organ damage show promise [50,51,52]. Health-related quality of life is often impaired in these children and requires longitudinal assessment. Additionally, the long-term effects on growth, dental health, and glandular reserve remain clinical concerns that warrant ongoing monitoring [13,51].

In conclusion, the prognosis for cSjD varies widely, ranging from relatively mild gland-predominant disease to multisystem, organ-threatening involvement in a minority of cases. Clinicians should adopt a long-term, multidisciplinary surveillance strategy, apply adult-derived risk markers judiciously, and enroll patients in pediatric registries to enhance the evidence regarding outcomes.

## 2. Case Presentation

A 17-year-and-7-month-old female presented to the General Pediatrics Clinic for evaluation of recurrent laterocervical and submandibular adenopathy and bilateral parotid swelling, more pronounced on the left side. The swellings were mildly tender, mobile, and firm. She denied having fever, weight loss, or other systemic symptoms at her presentation.

**Figure 1:**
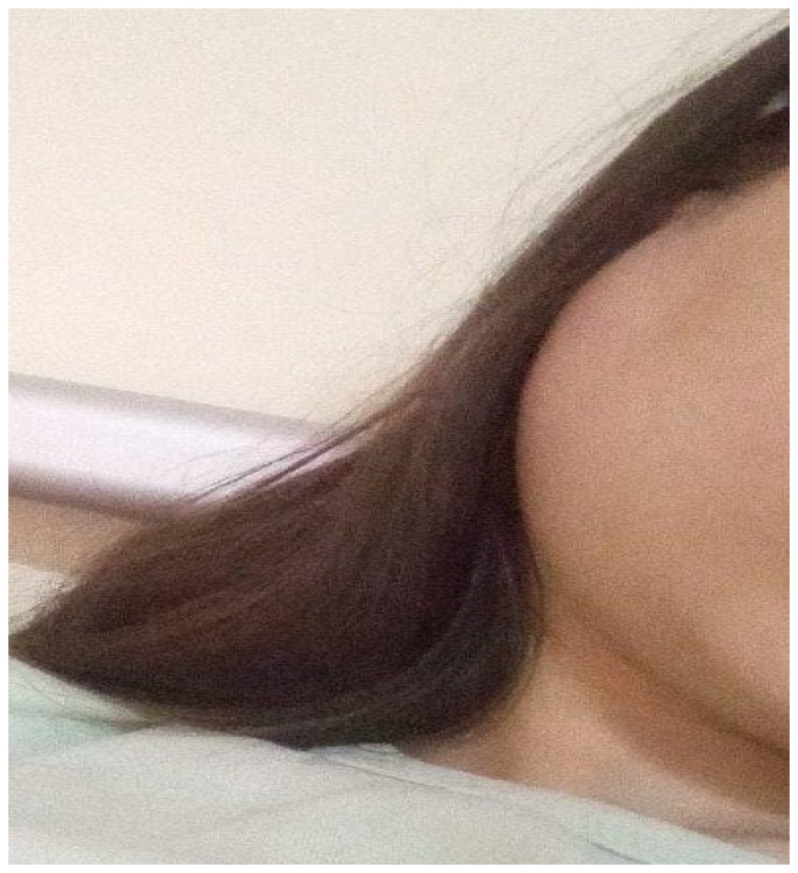
Right parotid gland swelling.

The patient had experienced multiple episodes of parotitis over the past seven years. Each episode was treated with antibiotic courses, providing only transient relief. Over time, she developed persistent xerostomia, intermittent bilateral parotid swelling, arthralgia affecting her knees, elbows, and ankles, and paresthesia predominantly in the lower limbs (Figure 1).

Her personal medical history was unremarkable, and she reported no prior chronic illness or medication use. Her family history was unavailable, as she was raised in foster care. Notably, she reported an inability to produce tears even when crying—a highly suggestive symptom of lacrimal hypofunction.

Physical examination revealed bilateral submandibular and laterocervical adenopathies, tender but mobile, with no signs of suppuration. Additional findings included bilateral cheilitis, a coated tongue, and cutaneous xerosis on the upper limbs. No hepatosplenomegaly or joint effusion was observed.

From the investigations previously performed in other hospitals, we report:

- Positive anti-Epstein–Barr virus (IgG) and anti-mumps virus (IgG) antibodies (six years prior).
- Soft tissue ultrasonography (six years prior) showing enlarged, hypervascular parotid glands and inflammatory cervical lymph nodes.
- Cranio-cerebral CT (performed approximately one month before presentation in our clinic) revealed calcifications in the choroid plexuses and pineal gland, as well as calcified atheromatous plaques in the carotid territory. The right frontal sinus was hypoplastic, and partial thickening of the mucosa of the right frontal and left maxillary sinuses was observed, as well as several ethmoidal cells on the right side, almost completely occupied by parafluid-density material.
- Cervical CT (performed approximately one month before presentation in our clinic): inflammatory lymphadenopathies, up to 12 mm.
- Soft tissue ultrasonography (performed approximately one month before presentation): parotid and submandibular glands replaced by hypoechoic nodular lesions and submandibular/jugular adenopathies up to 18 mm.

Our initial laboratory findings had shown leukopenia with lymphopenia, accelerated erythrocyte sedimentation rate (ESR), C-reactive protein (CRP) within normal limits, increased total protein levels, and mildly elevated transaminases, while renal and thyroid function parameters, serum amylase and lipase values, and glycemic profile were within normal limits. The peripheral blood smear did not reveal pathological changes. Serum protein electrophoresis and immunogram demonstrated hypergammaglobulinemia, with very high values of IgA and IgG fractions. Serological testing for infectious etiologies revealed no active infection: EBV IgG positive/IgM negative, CMV IgG positive/IgM negative, Toxoplasma gondii and Bartonella henselae IgG/IgM negative. QuantiFERON^®^ TB and RPR/VDRL tests were also negative (Table 1). Urinalysis was normal.

**Figure 2:**
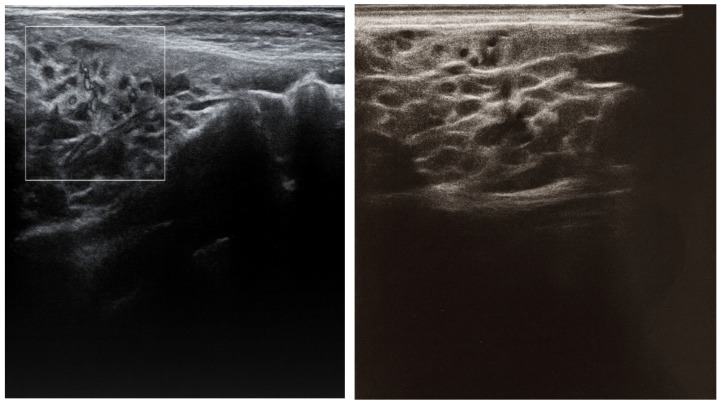
Submandibular gland ultrasound frames showing active inflammatory changes such as heterogeneous echotexture (“leopard-skin” or “snowstorm” appearance) and small hypoechoic foci, consistent with autoimmune sialadenitis.

Soft tissue ultrasound in our clinic showed multiple, non-confluent oval lymph nodes bilaterally in the laterocervical area, with hyperechoic centers and hypoechoic peripheries (largest: 12 × 17.6 mm in the left submandibular region). The parotid and submandibular glands appeared heterogeneous, containing numerous millimetric hypoechoic lesions (up to 2.2 mm) with intense Doppler vascularization. Intraparotid inflammatory lymph nodes were also noted (Figure 2).

Thyroid and abdominopelvic ultrasounds were unremarkable.

Chest X-ray showed accentuated bilateral hilar-basal interstitial thickening.

Thus, given the long history of recurrent parotitis episodes (despite antibiotic treatment), the clinical picture, including symptoms consistent with sicca syndrome and systemic manifestations (arthralgia, paresthesia), the presence of leukopenia with lymphopenia, and the detection of elevated levels of gammaglobulins (increased IgA and IgG), we considered the presence of an autoimmune disease, suggestive of a possible childhood-onset Sjögren’s disease.

With this suspicion, we continued the differential diagnosis by investigating the autoimmune component: serum complement fractions were within normal limits; the rheumatoid factor showed increased values; lupus cells and anti-double-stranded DNA antibodies were negative, while the patient tested positive for the anti-nuclear antibody (ANA). An extended ANA profile demonstrated high values of anti-SSA/Ro and anti-SSB/La antibodies.

**Figure 3:**
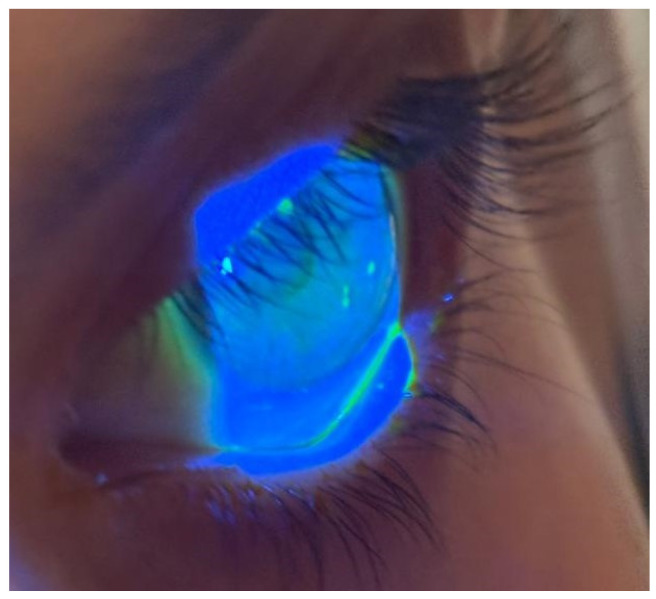
The image shows an ophthalmological exam performed with a slit lamp and fluorescein dye under blue (cobalt) light. The cornea appears illuminated in bright blue, with patches or dots of green fluorescence. These green areas represent spots where the fluorescein dye has penetrated the corneal epithelium—meaning tiny epithelial defects. Their irregular distribution, more pronounced in the lower part of the cornea, is typical of keratoconjunctivitis sicca—the ocular manifestation of SjD.

The ophthalmological evaluation identified a lusterless conjunctiva with a non-uniform tear film, a positive Schirmer test (1 mm in both eyes), a van Bijsterveld score of 4 in both eyes, and an abnormal fluorescein breakup time (FBUT) test (<6 s). These findings were consistent with severe dry eye disease (Figure 3).

**Figure 4:**
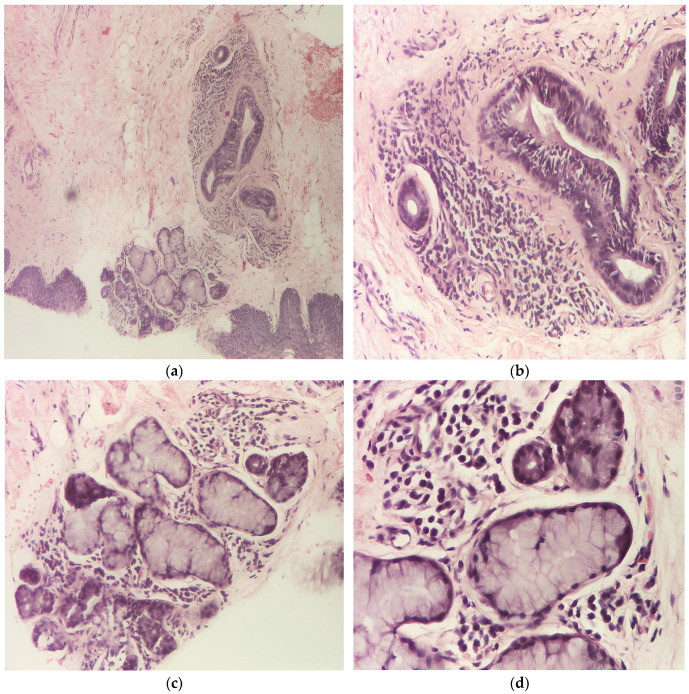
Histopathology of minor salivary gland biopsy in a patient with Sjögren’s disease: H&E sections at ×40 (**a**), ×100 (**b**,**c**), and ×200 (**d**) magnification demonstrate focal lymphocytic sialadenitis with acinar atrophy, periductal chronic inflammation, and two well-formed lymphocytic foci (>50 mononuclear cells) within the glandular parenchyma.

**Figure 5:**
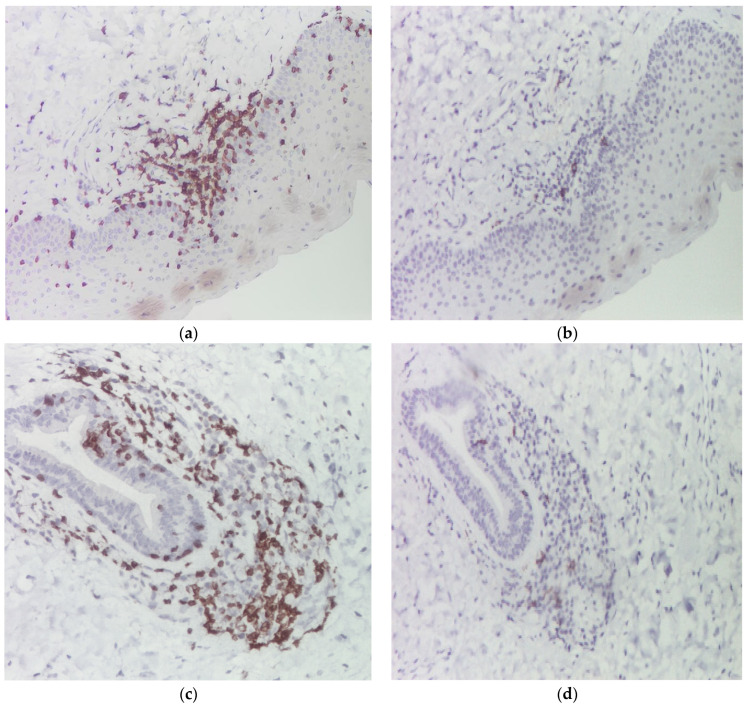
Immunohistochemistry staining for CD3 (**a**,**c**) and for CD20 (**b**,**d**), confirming the presence of mixed T- and B-cell infiltrates, but with a marked predominance of T lymphocytes. Only a minor population of B cells is present.

Unstimulated sialometry measured <1 mL of saliva in 15 min (<0.1 mL/min), indicating marked salivary gland hypofunction. A minor salivary gland biopsy from the lower lip demonstrates focal lymphocytic sialadenitis, the characteristic lesion of Sjögren’s disease. The glandular parenchyma contains dense, well-circumscribed aggregates of mononuclear inflammatory cells, predominantly small mature lymphocytes with scattered plasma cells. These lymphoid aggregates are observed both periductal and within the interstitial stroma, associated with acinar atrophy, ductal epithelial hyperplasia, and partial architectural distortion. Two well-formed lymphocytic foci (each focus contains > 50 mononuclear cells) are present within the glandular parenchyma per 4 mm^2^, corresponding to a calculated focus score of approximately 2 (Figure 4). This result is above the diagnostic threshold of ≥1, and in our clinical context, supports the diagnosis of SjD. No granulomas, vasculitis, or features of lymphoma are identified. Immunohistochemical staining confirmed the inflammatory infiltrate to be predominantly composed of CD3-positive T lymphocytes, with only scattered CD20-positive B cells. This T-cell–predominant pattern is characteristic of focal lymphocytic sialadenitis in SjD (Figure 5). No aberrant B-cell clustering or features suggestive of lymphoproliferative disorder were observed.

By correlating all these results and considering that our patient met the diagnostic criteria for pSS according to the 2016 ACR/EULAR classification (Table 2), we continued investigations to establish whether we were dealing with primary or secondary Sjögren’s disease.

The diagnostic workup excluded several potential autoimmune and metabolic conditions. Systemic lupus erythematosus was ruled out based on clinical presentation and a negative autoimmune panel. Thyroid ultrasonography and anti-thyroid peroxidase (anti-TPO) antibody levels were within normal limits, excluding autoimmune thyroiditis. A normal glycemic profile excluded type 1 diabetes mellitus, while anti-endomysium and anti-transglutaminase antibodies were negative, ruling out celiac disease. Given the clinical suspicion, a possible association of SjD with cryoglobulinemia was also evaluated; however, cryoglobulin levels were normal. Considering mild hepatocellular cytolysis, a comprehensive panel of autoantibodies related to autoimmune hepatitis was performed. All results were within normal limits except for anti-mitochondrial antibodies, which were detected at an equivocal level—insufficient to support a diagnosis of primary biliary cholangitis, an autoimmune condition frequently associated with SjD. IgG4-related disease was considered unlikely based on the patient’s clinical presentation, anti-SSA/anti-SSB positivity, and characteristic minor salivary gland histopathology.

Based on the clinical and laboratory findings, a diagnosis of primary SjD was established. According to the EULAR Sjögren’s Syndrome Disease Activity Index (ESSDAI), the total disease activity score was 10 points (Table 3).

The rheumatologist recommended methylprednisolone (16 mg/day) and HCQ (200 mg/day), while the ophthalmologist prescribed artificial tears for symptomatic management of dry eye. Methylprednisolone was initiated to achieve rapid control of systemic and glandular inflammatory activity, given the patient’s persistent parotid enlargement, elevated inflammatory markers, hypergammaglobulinemia, and evidence of active autoimmune disease. Hydroxychloroquine was introduced as a disease-modifying agent because of its established role in the management of SjD and its favorable safety profile in young patients. The treatment goals were to reduce disease activity, improve symptoms, prevent further glandular damage, and limit the development of additional systemic manifestations. Follow-up included ESR, CRP, immunoglobulin levels, salivary symptoms, ophthalmologic assessment, and ultrasound monitoring. After one month of therapy, reevaluation revealed significantly reduced cervical adenopathies. However, the patient developed a maculopapuloerythematous rash on the posterior thorax, pruritic and slightly pustular, attributed by the dermatologist to corticosteroid therapy. Topical fucidic acid and erythromycin/zinc acetate were prescribed, and systemic corticosteroid therapy was continued at tapering doses. At the time of reporting, the patient continues treatment with methylprednisolone (gradually tapered) and HCQ, showing a favorable clinical and biological evolution.

## 3. Discussion

cSjD remains one of the most challenging autoimmune conditions to diagnose in pediatric practice due to its rarity, heterogeneous presentation, and the absence of pediatric-specific classification criteria. Glandular manifestations are often absent at disease onset, whereas recurrent parotid swelling and systemic manifestations are more common. These features contribute to diagnostic delay in many pediatric patients. Consequently, many patients undergo years of repeated antibiotic therapy under the assumption of recurrent bacterial infection—as occurred in our patient—before an autoimmune etiology is considered.

In our case, the first manifestations appeared at the age of 10, with recurrent, painful parotid swelling and bilateral cervical adenopathy. Given the clinical context, these episodes were interpreted as viral or bacterial parotitis, and the patient received multiple courses of antibiotics over the years. This approach temporarily improved symptoms but did not address the underlying autoimmune process. This strategy contributed to a diagnostic delay of approximately 7 years, during which sicca symptoms, dry skin, and systemic manifestations (arthralgia, paresthesia) progressively developed.

Consequently, our patient exemplifies the chronic course of cSjD. The seven-year history of repeated episodes of parotid swelling unresponsive to antibiotics, together with persistent xerostomia and inability to cry, should have prompted earlier consideration of an autoimmune etiology. The inability to cry represents a striking and rarely reported clinical manifestation, directly reflecting lacrimal gland dysfunction and autonomic impairment of tear secretion, a clue often overlooked in pediatric evaluations. This symptom is particularly noteworthy given that emotional tear production is preserved even in many adults with SjD until advanced disease. Furthermore, an important diagnostic pitfall in this case was the persistent elevation of the erythrocyte sedimentation rate (ESR) in the absence of C-reactive protein (CRP) elevation. Over several years, this laboratory pattern was interpreted as evidence of a chronic or recurrent infectious process, leading to repeated antibiotic treatments. However, this discordance between elevated ESR and normal CRP is typical of chronic autoimmune diseases such as SjD, where elevated ESR reflects hypergammaglobulinemia rather than acute inflammation.

A particularly notable aspect of our patient is the presence of calcified carotid atherosclerotic plaques on cervical imaging. This raises important considerations regarding the relationship between chronic autoimmune inflammation and premature atherosclerosis in SjD.

Chronic systemic inflammation is a well-documented driver of accelerated atherosclerosis in several autoimmune diseases, including SjD. Studies in adult SjD populations show that patients have higher carotid intima-media thickness (cIMT) and a greater prevalence of subclinical atherosclerotic plaques compared to matched controls, independent of traditional cardiovascular risk factors [53,54,55]. For example, a large cohort study of 199 patients with primary SjD and 100 controls found a mean cIMT of 0.71 mm in SjD compared to 0.65 mm in controls. In age-stratified analyses (by decade), patients with SjD in their 50s had similar cIMT to controls in their 60s, and patients with SjD in their 60s had cIMT similar to controls in their 80s. These results strongly suggest that patients with SjD have earlier and more severe subclinical atherosclerosis (as measured by cIMT) than matched healthy controls, even after adjusting for classic cardiovascular risk factors [55]. A systematic review and meta-analysis (19 studies, 1625 participants) found that, compared to controls, patients with SjD had significantly higher carotid–femoral IMT (mean difference ~0.07 mm) and ~1.9-fold higher odds of plaques [54].

The mechanisms underlying premature vascular involvement in SjD are not yet fully elucidated but are thought to extend beyond traditional cardiovascular risk factors. Recent evidence suggests that chronic immune-mediated inflammation may contribute to endothelial dysfunction, arterial remodeling, and accelerated atherosclerosis through persistent cytokine activation and vascular injury. In addition, abnormalities in vascular function and macrovascular damage have been demonstrated in patients with primary SjD even after adjustment for conventional cardiovascular risk factors, supporting the concept of disease-specific vascular involvement. Although most available data originate from adult cohorts, these observations provide a biologically plausible framework linking chronic autoimmune inflammation with the development of subclinical atherosclerosis. Consequently, vascular abnormalities identified in younger patients with SjD may warrant particular attention, especially when alternative explanations are lacking [55,56,57].

In our patient, the finding of calcified carotid atheroma plaque may represent subclinical vascular involvement driven by chronic autoimmune and inflammatory processes rather than classic atherosclerotic risk factors, which were absent. The patient had no identifiable traditional cardiovascular risk factors. Her body mass index was 16.9 kg/m^2^ (weight 46 kg, height 165 cm), blood pressure was 115/58 mmHg, and lipid parameters were within normal limits (total cholesterol 158 mg/dL, LDL cholesterol 99 mg/dL, HDL cholesterol 46 mg/dL, triglycerides 100 mg/dL). In this context, the detection of calcified carotid atherosclerotic plaques raises the possibility of early vascular involvement associated with chronic autoimmune inflammation. The persistently elevated ESR, hypergammaglobulinemia, and autoantibody positivity reflect a milieu of immune activation that could contribute to endothelial dysfunction, oxidative stress, and vascular wall remodeling. Furthermore, the long diagnostic delay, during which the underlying SjD remained untreated, may have allowed ongoing low-grade inflammation to promote vascular damage. Literature suggests that longer disease duration correlates with increased IMT or plaque presence [53,54]. Recognizing vascular involvement in SjD—even in younger patients—is important. The presence of carotid plaque suggests a need for cardiovascular risk assessment. In SjD, standard cardiovascular risk calculators may underestimate risk because they do not incorporate autoimmune inflammation [58]. Thus, in patients with SjD (especially with systemic features or long-standing disease), early vascular imaging (such as carotid ultrasound) or non-invasive assessment of arterial stiffness may be considered. Lifestyle modification, control of modifiable risk factors, and vigilance for vascular complications should be integral parts of management. While adult studies on SjD and subclinical atherosclerosis are growing, there is a paucity of data on cSjD. To our knowledge, calcified carotid plaque in an adolescent with SjD represents a previously unreported finding. Although a direct causal relationship cannot be established, this observation raises the possibility that vascular involvement associated with chronic autoimmune inflammation may occur earlier than currently appreciated. This finding should be regarded as hypothesis-generating and highlights the need for further studies evaluating vascular health in children and adolescents with SjD. It also underscores the importance of multidisciplinary assessment (including rheumatology, cardiology, vascular surgery) and suggests that in SjD patients with long disease duration, vascular screening and early preventive strategies should be considered.

Another remarkable aspect of our case is the severity and completeness of the objective glandular dysfunction, supported by multiple converging diagnostic modalities. The patient exhibited severe keratoconjunctivitis sicca (Schirmer test: 1 mm), a critical reduction in unstimulated salivary flow (<1 mL/15 min), and characteristic ultrasonographic abnormalities with multiple intraparotid hypoechoic lesions. These findings, combined with strongly positive anti-SSA/Ro and anti-SSB/La antibodies and a compatible histopathological picture, led to a maximum cumulative score of 9 points according to the 2016 ACR/EULAR classification criteria.

Emerging evidence suggests that salivary autoantibodies, particularly anti-Ro60 and anti-Ro52, may represent promising non-invasive biomarkers for the early detection and stratification of SjD. Studies using luciferase immunoprecipitation systems demonstrated that anti-Ro60 autoantibodies can be detected in whole saliva in approximately 70% of patients with SjD, with high specificity comparable to serum testing, supporting their diagnostic relevance even at substantially lower concentrations than circulating antibodies. Furthermore, recent observational studies have shown that salivary anti-Ro60 antibodies may be present even in individuals with sicca symptoms who remain seronegative. Thus, salivary immunological profiling could help identify early or distinct disease phenotypes not captured by conventional serological assays. Although salivary anti-Ro60 testing was not available in our patient, incorporation of salivary autoantibody analysis into future diagnostic algorithms may improve early recognition of cSjD and facilitate non-invasive disease monitoring [59,60].

To our knowledge, this is the first reported case in the literature of a pediatric patient reaching the maximum possible score using these adult-derived classification criteria. Most published pediatric cases achieve lower scores because objective ocular tests and minor salivary gland biopsies often yield borderline or nonspecific findings, or because serological markers are absent at early disease stages. The complete positivity across all domains in our patient underscores the unusual degree of glandular destruction and immune activation occurring at a young age.

This raises several important considerations. First, the adult ACR/EULAR criteria, although widely used in pediatric cohorts due to the lack of dedicated pediatric standards, may underestimate or misclassify a substantial proportion of children with cSjD. Pediatric presentations often lack the objective dryness needed to meet adult criteria thresholds. In contrast, our patient’s severe sicca features demonstrate that cSjD can occasionally progress to a fully developed, adult-type phenotype, including systemic involvement and multiglandular dysfunction.

Recent efforts to improve recognition of cSjD have led to the development of pediatric-oriented diagnostic algorithms that place greater emphasis on recurrent parotitis, extraglandular manifestations, salivary gland ultrasonography, serological abnormalities, and histopathological findings rather than relying predominantly on sicca symptoms [34]. According to the recently proposed clinical diagnostic tool developed by the Childhood Sjögren Disease Workgroup, our patient would fulfill several hallmark features across multiple diagnostic pathways, including persistent parotid enlargement, anti-SSA positivity, characteristic salivary gland ultrasonography abnormalities, positive minor salivary gland biopsy findings, and objective ocular involvement. Therefore, the diagnosis in this case is supported not only by the adult 2016 ACR/EULAR classification criteria but also by emerging pediatric-specific diagnostic approaches, further strengthening the diagnostic certainty.

Compared with previously reported pediatric cohorts, our patient shared several characteristic features of childhood-onset Sjögren’s disease, including female sex, recurrent parotid involvement, anti-SSA positivity, hypergammaglobulinemia, and characteristic salivary gland imaging and histopathological findings [3,7,18]. However, the case also exhibited several unusual features. In contrast to many pediatric patients, in whom oral and ocular dryness may be absent or insufficiently developed to satisfy adult classification criteria at presentation [3,7,34], our patient demonstrated objective ocular and salivary gland involvement and fulfilled all major diagnostic domains included in the 2016 ACR/EULAR criteria. Furthermore, the coexistence of pronounced glandular disease, systemic immune activation, and vascular abnormalities distinguishes this case from the majority of previously reported pediatric cohorts [3,7,18]. These findings suggest a disease phenotype that closely resembles adult primary Sjögren’s disease despite the young age at diagnosis and underscore the marked clinical heterogeneity of childhood-onset disease.

A limitation of the present report is the relatively short follow-up period available within the pediatric setting. Following diagnosis, the patient was transitioned to adult rheumatology care after reaching the age of 18 years, limiting access to long-term follow-up data regarding treatment response, disease progression, and preservation of salivary gland function. Consequently, the long-term clinical and imaging outcomes of this case could not be systematically assessed within the scope of the present report.

## 4. Conclusions

This case illustrates the diagnostic challenge in cSjD. The absence of early pathognomonic signs, together with a predominantly infectious-appearing phenotype, explains the frequent misdiagnosis and delay in recognition. The consequences of such a delay include unnecessary antibiotic exposure, prolonged morbidity, and the risk of progressive glandular and extraglandular involvement. Early diagnosis is essential not only to improve the quality of life by addressing sicca symptoms, but also to monitor and prevent systemic complications, including pulmonary, renal, and neurological manifestations, as well as the long-term risk of lymphoma.

To our knowledge, this is the first reported pediatric case simultaneously demonstrating a maximum ACR/EULAR classification score together with objective evidence of premature carotid atherosclerotic involvement in the absence of traditional cardiovascular risk factors. These findings support the concept that cSjD may present with a fully developed glandular phenotype and vascular manifestations earlier than previously recognized. By documenting this association in an adolescent patient, our report contributes new evidence supporting the systemic and potentially vasculopathic nature of cSjD and underscores the importance of earlier diagnostic suspicion in children with recurrent parotitis and persistent inflammatory abnormalities.

## Figures and Tables

**Table 1:** Laboratory findings of the patient.

Test	Result	Reference Range	Units
WBC	**3.24**	4.5–13	×10^3^/µL
Neutrophils	1.93	1.8–8	×10^3^/µL
Lymphocytes	**0.82**	1.5–6.5	×10^3^/µL
Platelets	165	150–400	×10^3^/µL
Hemoglobin	12.4	11.7–15.3	g/dL
Hematocrit	35.5	34–44	%
CRP	1.3	0–5	mg/dL
ESR	**95**	0–12	mm/h
ALT	**42**	5–35	U/L
AST	34	10–34	U/L
GGT	20	7–22	U/L
Total bilirubin	0.54	0.3–1.2	mg/dL
Unconjugated bilirubin	0.38	0.1–0.75	mg/dL
Conjugated bilirubin	0.16	0.1–0.6	mg/dL
LDH	132	100–250	U/L
Alkaline phosphatase	73	46–116	U/L
BUN	25	19.26–49.22	mg/dL
Creatinine	0.79	0.6–1.2	mg/dL
Total protein	**101.2**	57–82	mg/dL
Amylase	72	32–117	U/L
Lipase	39	18–47	U/L
Ferritin	24	15–150	µg/L
Glycemia	85	60–100	mg/dL
HbA1c	5.4	<5.7	%
C3	116	83–193	mg/dL
C4	14	12–36	mg/dL
Rheumatoid factor	**17.81**	0–14	UI/mL
Anti-dsDNA	5.61	<25	UI/mL
IgA	**507.6**	71–335	mg/dL
IgG	**3557**	684–1580	mg/dL
TSH	2.37	0.47–3.41	µUI/mL
fT4	0.96	0.89–1.37	ng/dL
ATPO	0.57	0–5.61	UI/mL
Anti-SSA antibodies	**positive (SS-A 52–64; SS-A 60–7.6)**	<0.3	kU/L
Anti-SSB antibodies	**positive (137)**	<0.3	kU/L

WBC—white blood cell count; CRP—C-reactive protein; ESR—erythrocyte sedimentation rate; ALT—alanine aminotransferase; AST—aspartate aminotransferase; GGT—gamma-glutamyl transferase; LDH—lactate dehydrogenase; BUN—blood urea nitrogen; HbA1c—glycated hemoglobin; C3/C4—complement components 3 and 4; dsDNA—double-stranded DNA; IgA/IgG—immunoglobulin A/G; TSH—thyroid-stimulating hormone; fT4—free thyroxine; ATPO—anti-thyroid peroxidase antibodies; SSA/SSB—anti-Ro/anti-La antibodies.

**Table 2:** Diagnostic Scoring of the Patient According to the 2016 ACR/EULAR Criteria for pSS.

Criteria	Score
Minor salivary gland biopsy with focal lymphocytic sialadenitis and Focus Score ≥ 1	3 points
Anti-SSA (Ro) antibodies positive	3 points
Ocular staining score ≥ 5 (or van Bijsterveld ≥ 4)	1 point
Schirmer’s test ≤ 5 mm/5 min	1 point
Unstimulated whole salivary flow rate ≤ 0.1 mL/min	1 point
Total score	9 points

Classification requires a total score ≥ 4. The patient reached a score of 9, fully meeting the 2016 ACR/EULAR criteria for pSS.

**Table 3:** ESSDAI score of the patient.

Domain	ESSDAI Score
lymphadenopathy ≥ 1 cm in any nodal region	4 points
arthralgia in hands, wrists, ankles and feet accompanied by morning stiffness	2 points
lymphopenia with 500 < lymphocytes < 1000/mm^3^	2 points
presence of cryoglobulinemia and/or hypergammaglobulinemia or high IgG level > 20 g/L	2 points
Total ESSDAI score	10 points

ESSDAI: EULAR Sjögren’s Syndrome Disease Activity Index. Scoring categories: 0 = no activity, 1–4 = low activity, 5–13 = moderate activity, ≥14 = high activity. This patient’s score of 10 corresponds to moderate systemic disease activity.

## Data Availability

All data supporting the findings of this study are contained within the article. Additional information can be provided by the corresponding author upon reasonable request.

## Abbreviations

The following abbreviations are used in this manuscript:

ACR	American College of Rheumatology
ANA	Antinuclear Antibody
BAFF	B-cell Activating Factor
CBC	Complete Blood Count
CFTR	Cystic Fibrosis Transmembrane Conductance Regulator
cIMT	Carotid Intima-Media Thickness
CMV	Cytomegalovirus
CRP	C-reactive protein
cSjD	Childhood-Onset Sjögren’s Disease
CT	Computed Tomography
DNA	Deoxyribonucleic Acid
dRTA	Distal Renal Tubular Acidosis
EBV	Epstein–Barr Virus
ESR	Erythrocyte Sedimentation Rate
ESSDAI	EULAR Sjögren’s Syndrome Disease Activity Index
EULAR	European League Against Rheumatism
FBUT	Fluorescein Break-Up Time
HCQ	Hydroxychloroquine
HLA	Human Leukocyte Antigen
HSV	Herpes Simplex VIrus
IFN	Interferon
IgA	Immunoglobulin A
IgG	Immunoglobulin G
IgM	Immunoglobulin M
IL	Interleukin
IMT	Intima-Media Thickness
MALT	Mucosa-Associated Lymphoid Tissue
mSGB	Minor Salivary Gland Biopsy
MRI	Magnetic Resonance Imaging
PCR	Polymerase Chain Reaction
pSS	Primary Sjögren Syndrome
RF	Rheumatoid Factor
RPR	Rapid Plasma Reagin
SGUS	Salivary Gland Ultrasonography
SjD	Sjögren’s Disease
SSA	Anti-SSA (Ro) Antibodies
SSB	Anti-SSB (La) Antibodies
TB	Tuberculosis
TPO	Thyroid Peroxidase
UHF	Ultrahigh Frequency
US	Ultrasound
VDRL	Venereal Disease Research Laboratory
